# Far transfer of retrieval-practice benefits: rule-based learning as the underlying mechanism

**DOI:** 10.1186/s41235-024-00598-y

**Published:** 2024-10-08

**Authors:** Bertram Opitz, Veit Kubik

**Affiliations:** 1 School of Psychology, University of Surrey, Guildford, UK; 2 Present Address: Department of Psychology, Brandenburg Medical School, Neuruppin, Germany; 3 Department of Psychology, FernUniversität in Hagen, Hagen, Germany; 4 Department of Psychology IV, Julius Maximilian University of Würzburg, Würzburg, Germany

**Keywords:** Testing effect, Retrieval practice, Far transfer, Artificial language, Rule-based learning, Inductive learning

## Abstract

Benefits of self-testing for learning have been consistently shown for simple materials such as word lists learned by rote memorization. Considerably less evidence for such benefits exists for complex, more educationally relevant materials and its application to new situations. The present study explores the mechanisms underlying this transfer. To this end, a typical retrieval-practice-effect paradigm was applied to foster the learning of an artificial language. Participants either repeatedly studied grammatically correct exemplar sentences of the artificial language or engaged in a cloze test as the interim test after learning. To assess far transfer, participants in both groups of restudy and retrieval practice engaged in a grammaticality judgment test after a delay of 5 min and 1 week. In addition, participants in both groups completed a final memory test (i.e., a cloze test identical to the initial test) 1 week after learning. In addition to a long-term memory benefit of retrieval practice, results revealed also a retrieval-practice benefit in the far-transfer test after the 1-week delay. The findings further support the view that far transfer is supported by learning the underlying grammatical rules as opposed to memorizing the material. Thus, retrieval practice is also effective for fostering learning of complex materials and, even more importantly, for promoting transfer of learning—a crucial goal in modern educational practices.

## Introduction

Interest in the benefits of self-testing for subsequent memory has increased dramatically in recent years due to the important implications for student learning and educational practice (Kubik et al., 2021; for an overview, see McDermott, 2021; for a meta-analysis, see Adesope et al., 2017; Yang et al., 2021). The robust finding emerging from many studies is that taking tests (i.e., retrieval practice) enhances later retention more than additional study opportunities, even when no feedback is given (Carrier & Pashler, 1992; Halamish & Bjork, 2011; Kubik et al., 2020; Roediger & Karpicke, 2006a). For instance, in a seminal study (Wheeler et al., 2003), participants studied a 40-word list either five times (repeated study condition) or one time with four consecutive recall tests (retrieval-practice condition). Results showed that repeated study produced a higher level of recall in an immediate test 5 min after the learning phase than retrieval practice. However, due to an accelerated forgetting rate for repeated study, this restudy advantage was reversed 1 week later into a long-term benefit in favor of repeated testing in the retrieval-practice condition. This finding of the so-called testing effect or retrieval-practice effect^1^ on memory is robust, with effect sizes of Hedges’ *g* = 0.50–0.63 (e.g., Roediger & Karpicke, 2006b; for meta-analyses, see Adesope et al., 2017; Rowland, 2014).

These studies converge on the idea that the testing effect is not due to additional exposure to the material but tied to the act of memory retrieval itself. There are multiple theories that account for the testing effect. For example, retrieval effort theories suggest that retrieval practice provides a desirable difficulty (Bjork & Bjork, 2020) that, for example, enhances learning through semantic elaboration of existing memory traces (e.g., activating associates of the target; Carpenter & DeLosh, 2006; Carpenter, 2009, 2011) or by multiplying the number of retrieval routes (McDaniel & Fisher, 1991). Another explanation relates to the idea that the testing effect results from the processing of episodic context information (such as time or location of the memoranda) that helps to reduce interference (Szpunar et al., 2008; see also Lehman et al., 2014) and provide additional, distinctive cues to recover the target information in the future (Karpicke et al., 2014).

Overall, the focus of most previous studies on retrieval practice has been on simple materials that could be learned by rote memorization like word lists or simple factual information (see Carpenter et al., 2022; Yang et al., 2021; for a discussion, see Adesope et al., 2017; Schwieren et al., 2017). Rote memorization is characterized by sheer repetition of facts with no or little understanding of the content being learnt. While rote memorization allows to recall basic facts and helps develop foundational knowledge, it does not allow for a deeper understanding of a subject and, most of all, it does not connect new knowledge with previous knowledge (Tan, 2014). In relation to educationally relevant, more complex materials, findings seem to be more heterogeneous. While few studies report that the retrieval-practice effect may decrease or even disappear when the complexity of learning material is very high (see van Gogh & Sweller, 2015), there is a growing body of evidence that retrieval practice is also effective with educationally relevant, more complex materials (e.g., Endres & Renkl, 2015; Heitmann et al., 2018, 2021; for a discussion of these inconsistencies, see Karpicke & Aue, 2015; Rummer & Schweppe, 2022) and in authentic educational contexts (at the university: e.g., Greving & Richter., 2022; Heitmann et al., 2022; for a meta-analyses, Adesope et al., 2017; Schwieren et al., 2017; in school settings: Karpicke et al., 2016; for a meta-analysis, see Agarwal et al., 2021). The benefits of retrieval practice have been demonstrated for a number of different test formats, like assessing knowledge in short-answer or multiple-choice tests (Agarwal et al., 2008; Greving & Richter, 2018, 2022; Roelle et al., 2022; for a meta-analysis, see Adesope et al., 2017; Schwieren et al., 2017).

Crucially, educational contexts require the learner to apply knowledge acquired in a specific learning context to various quite different test contexts (see e.g., Barenberg et al., 2021; Richter et al., 2022). For example, the format of formative assessments to foster learning (e.g., a multiple-choice test) often differs from the format of summative tests assessing learning at the end of an instructional unit (e.g., open-ended questions; Roelle et al., 2022) or must be integrated with new information to answer inference questions (Pan & Pickard, 2018). Hence, the transfer of learning to new situations is a central requirement in education (Richter et al., 2022). Depending on the difference between the initial learning context and the test context, this application of knowledge is often dichotomized into near and far transfer, that is, transfer to similar and highly overlapping situations (like a change in test format) and transfer to situations substantially differing from the original learning context (e.g., requiring the application of learning to new but conceptually related information), respectively (c.f., Barnett & Ceci, 2002; Pan & Pickard, 2018). Note that according to Barnett and Ceci (2002) the divide of transfer into near and far transfer should be considered on various dimensions like knowledge domain, situational context, task demands, or acquired knowledge. Regarding the knowledge domain of transfer, the application of knowledge in the same knowledge domain is considered as near transfer, while the application in a different domain is considered far transfer. This view is also adopted in most studies on cognitive training (e.g., Melby-Lervåg & Hulme, 2013). However, with respect to the content dimension of the acquired knowledge, the retrieval of learned information based on superficial features is considered near transfer, while the generation and application of a general principle, that could be characterized as a deeper, structural, or causal understanding, rather reflect far transfer (Barnett & Ceci, 2002). Examples for the latter notion of far transfer include inductive learning involving the abstraction of general characteristics among multiple examples. For instance, in a math class, students study a series of addition or multiplication examples to induce a general principle of addition and multiplication; medical students study a series of symptoms across several patients to find general patterns of abnormalities characteristic of a certain disease; or students listen to a text in a foreign language to learn about grammatical structures in that language. For these examples, as for inductive learning in general, it is often not sufficient to remember and retain specific instances. Learners must induce general patterns or rules from the studied examples and generalize this knowledge to other new examples (Holland et al., 1989).

Evidence available to date seems to suggest larger positive effects of retrieval practice for near transfer and smaller positive or sometimes negative effects for far transfer (Barenberg et al., 2021; Brunyé et al., 2020; Cho & Powers, 2019; Jacoby et al., 2010; Kang et al., 2023; Pan & Pickard, 2018). Several studies report some transfer to novel exemplars of learned categories (Cho & Powers, 2019; Jacoby et al., 2010; Kang et al., 2023). Notably, in all these studies novel exemplars were visually similar to previously encountered exemplars of the same category, for instance, by sharing a radical of Chinese words (Cho & Powers, 2019), or surface features of rocks (Kang et al., 2023). In line with the taxonomy for transfer proposed by Barnett and Ceci (2002), this could be considered as near transfer because the knowledge domain and the learned representations are highly similar.

In other studies, transfer was induced by changing task requirements in the final test compared to the initial retrieval-practice task (Barenberg et al., 2021; Brunyé et al., 2020). While one study (Brunyé et al., 2020) demonstrated no benefits of retrieval practice for far transfer of spatial knowledge to pointing or navigation tasks performed from an alternate perspective, Barenberg and colleagues (2021) were able to demonstrate transfer of retrieval-practice effects on English vocabulary learning. Across three different transfer tasks, transfer varied by the degree of application of the acquired material (e.g., sentence-generation task) and yielded significant but smaller effect sizes compared to a non-transfer condition, in which the final test conformed with the interim test (i.e., cued recall).

In addition, transfer of retrieval-practice effects has also been demonstrated for forward transfer in category learning (Lee & Ahn, 2018; Lee & Ha, 2019; Yang & Shanks, 2017). In these studies, participants learned the painting styles of different artists before and after an interim-testing session through associative learning. Again, the group of participants receiving an interim cued-recall test was more successful in a final test requiring the classification of new paintings by the same artists than the group with no interim testing.

Despite this intriguing demonstration of the effectiveness of retrieval practice for transfer to novel exemplars in category learning tasks, it remains unknown whether retrieval practice enhances learning with more complex material requiring abstraction beyond the level of discrete categories. Furthermore, the mechanisms underlying near and far transfer are not fully understood. Particularly far transfer, that is, the application of the acquired knowledge to different content in varying contexts, seems to require complex inductive learning (i.e., the abstraction of general characteristics beyond a specific context) involving various (rule- and similarity-based) mechanisms.

Second language acquisition is an example of such complex inductive learning as the acquisition of grammatical knowledge in a foreign language has been widely assumed to involve the acquisition of abstract structural rules (Chomsky, 1965). Due to the complexity of natural languages, researchers have thus turned to artificial languages as a means of obtaining better control over several characteristics of learning, for example, syntactic or phonological processing, in isolation from semantic influence (e.g., Gomez & Gerken, 2000; Mueller, 2006; Opitz & Kotz, 2012; Uddén & Männel, 2018). For instance, artificial languages have been generated to share many fundamental aspects of natural language (Fitch & Friederici, 2012). Despite being capable of generating an infinite set of outputs (“grammars”) in principle, such artificial languages vary in their generative power depending on the rule system involved. The weakest system of local organizational rules is specified by transition probabilities between neighboring elements. Stronger systems, required to process any natural language, in addition to simply concatenating elements, can embed sequences within other sequences and thus create complex hierarchical structures and long-distance dependencies (Fitch & Friederici, 2012; Opitz & Friederici, 2007). It has been recently argued that such artificial grammars could be acquired by different learning mechanisms: similarity- and rule-based mechanisms (Hauser et al., 2012; Opitz & Friederici, 2004; Opitz & Hofmann, 2015). The similarity-based mechanism of learning suggests that individuals learn the grammar by judging the similarity of novel stimuli in reference to a memory representation of previously learned stimuli, while the rule-based mechanism refers to a computational construct of mental representations capturing abstract regularities of several grammatical stimuli (Opitz, 2010; Opitz & Hofmann, 2015). Particularly the latter mechanism requires active organization of the input, rather than passive absorption. Given that retrieval is considered a constructive process (cf. Whittlesea & Wright, 1997), retrieval practice might, therefore, foster such active organization.

This distinction between rule-based and similarity-based learning is akin to theoretical accounts of the benefits of retrieval practice on near and far transfer of learning that could be divided into two perspectives: abstractionist models and similarity-based models (cf. Barnett & Ceci, 2002). Abstractionist perspectives assume that learning of underlying general principles (e.g., rules, operations, procedures, etc.) can facilitate far transfer, while similarity-based accounts propose that transfer is determined by the number of overlapping elements between the learning and transfer context (Barenberg et al., 2021; Pan & Pickard, 2018). Thus, similarity-based accounts imply that mainly near transfer is facilitated by retrieval practice.

The aim of the present study is to investigate the effectiveness of retrieval practice for complex material that goes beyond mere memorization but requires transfer of knowledge. To this end, we let students learn the grammatical rules of the artificial language *BROCANTO* (Friederici, et al. 2002; see Method section) and probe students’ acquired grammatical knowledge with both memory and transfer tests after a delay of 1 week. The memory test uses the same task as used for retrieval practice, but compares previously learned sentences of the artificial language with new sentences. In contrast, the transfer test employs a different task requiring the application of abstract principles (i.e., the rule system of BROCANTO) of the learned artificial language as well as material never encountered before. In line with the taxonomy for transfer proposed by Barnett and Ceci (2002), this transfer task is considered as assessing far transfer as both the content (rule knowledge) and the context (knowledge domain) are considerably different from the initial learning situation. Furthermore, we will test which of the two learning mechanisms of rule-based learning versus similarity-based learning are driving potential far-transfer benefits of retrieval practice. Based on the transfer test of grammaticality judgments, we estimate specific parameters reflecting rule-based and similarity-based learning mechanism from the receiver operating characteristic (ROC) of each participant (for more information, see Methods section; see also Opitz & Hofmann, 2015).

Based on prior findings on transfer and retrieval practice evoking rule-based and similarity-based mechanisms, we predict a retrieval-practice transfer effect in complex inductive learning in addition to a retrieval practice effect in long-term retention. Thus, we would predict similar performance in the transfer test after a short delay (5 min) and long delay (1 week) for retrieval practice, while repeated study should result in a decline in performance after 1 week. Furthermore, we hypothesize that far transfer is fostered by a rule-based learning mechanism; that is, we predict a benefit of retrieval practice over restudy for rule-based knowledge but not similarity-based knowledge.

## Methods

### Participants

A power analysis (R version: 4.0.2., package: pwr) for a general linear model indicated a sample size of 100 participants to detect small to medium effect sizes (${\upeta }_{\text{p}}^{2}$ = 0.04) based on previously reported effect sizes for transfer effects (e.g., Barenberg et al., 2021, Fig. 2) with a power of 0.9. To account for unknown attrition rates in multisession online studies, 145 participants with no prior knowledge of the artificial language BROCANTO were recruited by volunteer sampling through advertisements on university notice boards and social media networks (Instagram and Facebook). The post specified that only participants aged 18 years or older and who had not been diagnosed with dyslexia were able to take part in the study. Of the 145 initially recruited volunteers, 44 participants were native speakers of English and 101 were native speakers of German. If eligible, participants were offered course credits in partial fulfillment of study requirement.

Participants were randomly allocated to either the retrieval-practice group (*n* = 72) or restudy group (*n* = 73). While 117 participants (58 and 59 participants in the retrieval-practice and restudy group, respectively) completed both sessions, 28 participants dropped out after the first session. In this final sample, 79 identified as female, 37 as male, and one participant identified as non-binary. The mean age was 28.6 years (age range = 18–67 years).

### Materials

**Fig. 1:**
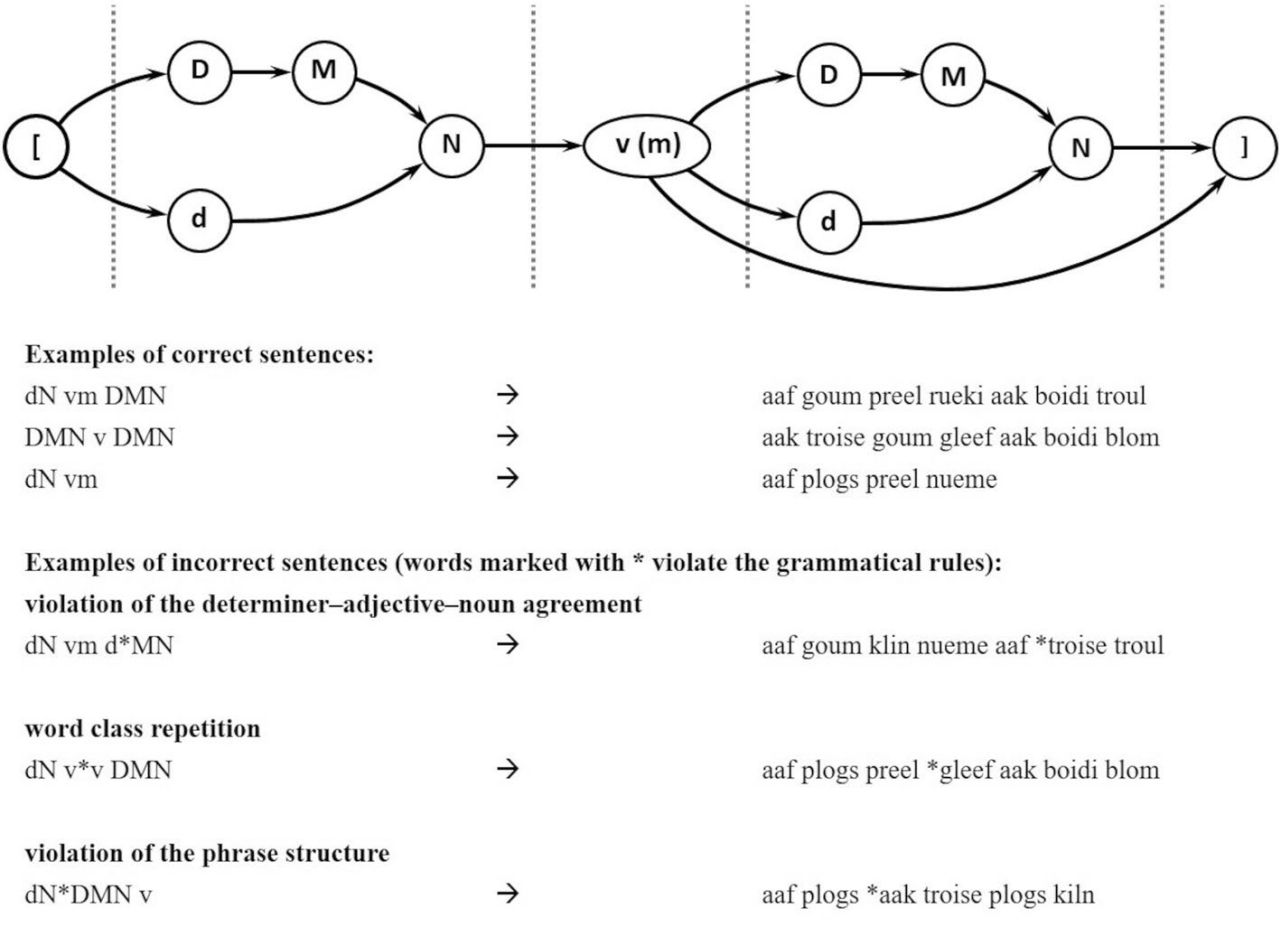
Schematic illustration of the rules of the artificial grammar language BROCANTO (top) and examples of grammatically correct and incorrect sentences. The examples of grammatically incorrect sentences depict the three violation types: violations of the determiner–adjective–noun agreement, word class repetitions, and violations of the phrase structure

The material was formed according to the artificial language BROCANTO (Opitz & Hofmann, 2015, see Fig. 1). The vocabulary comprised fourteen words from five different word categories: nouns (N), verbs (v), determiners (D, d), adjectives (M), and adverbs (m). Word categories contained two to four members and were identifiable by particular vowels (e.g., u or o indicating a noun; e or i = verb). Each sentence of the artificial language built from this vocabulary, contained three to eight words, and represented a subject–verb–[object] structure. The subject and the optional object of a sentence were a noun phrase (NP) composed of a determiner (D, d), an adjective (M), and a noun (N). The verb phrase (VP) consisted of a verb (v) and an optional adverb (m). A total of 160 sentences (70 sentences for training and 90 sentences for testing) were formulated according to these rules. Another 90 sentences were grammatically incorrect, that is, they contained a severe syntactic violation. Three types of violations were constructed covering a wide range of possible violations. These include violations of the phrase structure (i.e., an NP was presented instead of VP), violations of the determiner–adjective–noun agreement, and word class repetitions (for examples, see Fig. 1; for a detailed description, see Opitz & Friederici, 2003).

The task was programmed in PsychoJS (Version 2021.1.2) and administered through Pavlovia (pavlovia.org).

### Procedure

The study procedures were reviewed by and received a favorable ethical opinion from local ethics review boards (Reference Number: FHMS 20-21_085 and EA_232_2020). Before enrolling in the study, each participant was provided with detailed information about the study and was asked to give consent before starting the task.

For the first session, participants were randomly allocated to either the retrieval-practice group or the restudy group. Participants in both practice types were exposed to 10 correct examples of BROCANTO. They were instructed to use the examples to figure out how BROCANTO *works* (i.e., to attempt learning the grammatical rules of BROCANTO). The duration of sentence presentation varied with the length of the sentence; for every word one second was added to a base duration of one second. For example, a sentence with four words was presented for five seconds and a sentence with eight words was presented for nine seconds. This allowed sufficient time to read and evaluate longer sentences while ensuring optimized presentation rate. Participants in the restudy group were then shown the same 10 sentences again, while participants in the retrieval-practice group were given a cloze test. In this cloze test, participants were shown the same 10 sentences one by one. In each sentence one word apart from anchor words (i.e., the first or the last word in a sentence) was replaced by a dashed line. Underneath the sentence, the cloze word (i.e., the word that originally completed the sentence) was presented alongside another word that would not yield a grammatically correct sentence. The side of presentation (left or right) of these two candidate cloze words was counterbalanced across trials. Participants were asked to indicate which of the words would lead to a grammatically correct sentence by pressing the keys *1* or *2* on a computer keyboard. No feedback was provided. This procedure was repeated six times until both groups were exposed to 70 different grammatically correct sentences.

Once participants completed this practice phase, they were admitted to a final transfer test of grammaticality after a delay of 5 min. Participants were shown a new set of 90 sentences, half of which violated the grammatical rules of BROCANTO. Each sentence was displayed for a duration adjusted for the length of the sentence in the same way as during learning. Each sentence was followed by a fixation cross shown for one second. Participants were asked to judge the grammaticality of the sentence according to the grammar rules they just learned. Grammaticality judgments were given on a 6-point scale allowing participants to additionally state the confidence of their judgments, ranging from 1 (*surely grammatically correct*) to 3 (*rather grammatically correct*) given with one hand for the supposedly correct sentences and from 4 (*rather grammatically incorrect*) to 6 (*surely grammatically incorrect*) given with the other hand for the supposedly incorrect sentences. The assignment between hand and grammaticality judgment was counterbalanced across participants. Again, no feedback was provided.

Participants were asked to provide their email addresses to send them the link for the second session after 1 week. This second session was identical for all participants. They were first asked to complete the cloze test for 70 sentences. To differentiate between retrieval-practice effects on memory of old materials and on transfer to novel materials, half of these sentences were taken from the first learning session (old sentences), while the other half of the sentences were not seen before (new sentences). After the cloze test, participants were presented with the same transfer grammaticality test as in the first session, however, it included 90 new sentences that the participants have never seen before. Half of these new sentences were grammatically correct and the other half violated the grammatical rules of BROCANTO. Participants were asked to judge the grammaticality of these sentences using the same response options and assignments as during the first learning session. This grammaticality test can be considered as a test of far transfer because it not only involves a different test format (grammaticality judgment) compared to the format of the initial retrieval practice (cloze test) but also requires the application of learned knowledge to novel material.

### Data analysis

Raw data were processed in R Studio (Version 1.3.959; R version: 4.0.2., with packages *tidyverse* and *psycho*) in order to extract the data. Data were first screened for compliance with the instruction. Non-compliance was assumed when a participant’s discrimination between grammatically correct and incorrect sentences was not significantly different from guessing (*d*′ < 0.5) and their mean response time (from the beginning of the presentation of a sentence) was less than 1 s at the same time. It was deemed that this reaction time is too short to read the sentence and make an informed grammaticality decision. Participants exhibiting this response pattern were excluded from all analysis. In contrast, poor discrimination performance and a mean response time larger than 1 s were considered as compliant responding indicating that a participant had legitimately not learned the grammatical rules. Based on the combined criterion, 21 participants (nine from the retrieval-practice group and 12 from the restudy group) were excluded from all analyses.

For the cloze test, the proportion of correct responses was calculated separately for previously seen, old sentences, and new sentences. For the analysis of grammaticality judgments, responses were collapsed across several confidence levels to represent grammatical responses (i.e., a 1, 2, or 3 confidence rating) or non-grammatical responses (i.e., a 4, 5, or 6 confidence rating), respectively. This dichotomization corresponds to the instructed meaning of the numbers (see Procedure; for a similar approach, see Brod & Opitz, 2012; Opitz & Hofmann, 2015). Mean endorsement rates (i.e., mean proportion of “grammatical” responses) for grammatically correct and incorrect sentences were subjected to a repeated-measure ANOVA with the within-subject factors sentence type (grammatical vs. non-grammatical sentences) and delay (5 min vs. 1 week) and the between-subject factor practice type (retrieval practice vs. restudy). Subsidiary ANOVAs were performed to follow up significant interactions.

In addition, receiver operating characteristics (ROC) were calculated for each participant based on the respective response profile (see Opitz & Hofmann, 2015). For this analysis, true positives were defined as the correct identification of grammatical sentences (i.e., “gr” | gr; “grammatical” answer to a grammatically correct sentence) and false positives as a “grammatical” answer to grammatically incorrect sentences (i.e., “gr” | non-gr). Empirical ROC points were then constructed by cumulating the mean true and false positive rates separately across levels of confidence. Thus, the first point on the ROC represents the performance for the first confidence level (i.e., “surely grammatically correct” responses). This procedure was continued for each successive level of confidence ending with the “surely grammatically incorrect” responses. A hybrid ROC model, based on the assumption that grammaticality judgments in the transfer test can be independently based on both, learned rule knowledge and the assessment of the similarity of a test sentence to a memory representation, was fitted to the empirical data. The basic assumption is that rule knowledge is reflected in a high-threshold process, that is, either the rule applies or it does not. In contrast, for similarity-based judgments test items are assessed via a Gaussian equal-variance signal-detection process. The model equation reflects the assumption that a true positive response (a “grammatical” answer to a grammatically correct sentence) occurs when a grammatical item is endorsed either by rule knowledge or is endorsed as grammatical on the basis of similarity given that there is no rule knowledge. Likewise, a non-grammatical item is incorrectly endorsed as grammatical solely on the basis of similarity (for a detailed outline of the assumptions, a description of procedures, and the mathematical models, see Opitz & Hofmann, 2015). Thus, fitting the model equation to the empirical data yields two parameter estimates reflecting rule-based and similarity-based learning. These parameter estimates were subjected to a repeated-measures ANOVA the within-subject factors parameter estimate (rule vs. similarity), delay (5 min vs. 1 week), and the between-subject factor practice type (retrieval practice vs. restudy).

## Results

### Retrieval-practice performance

**Fig. 2:**
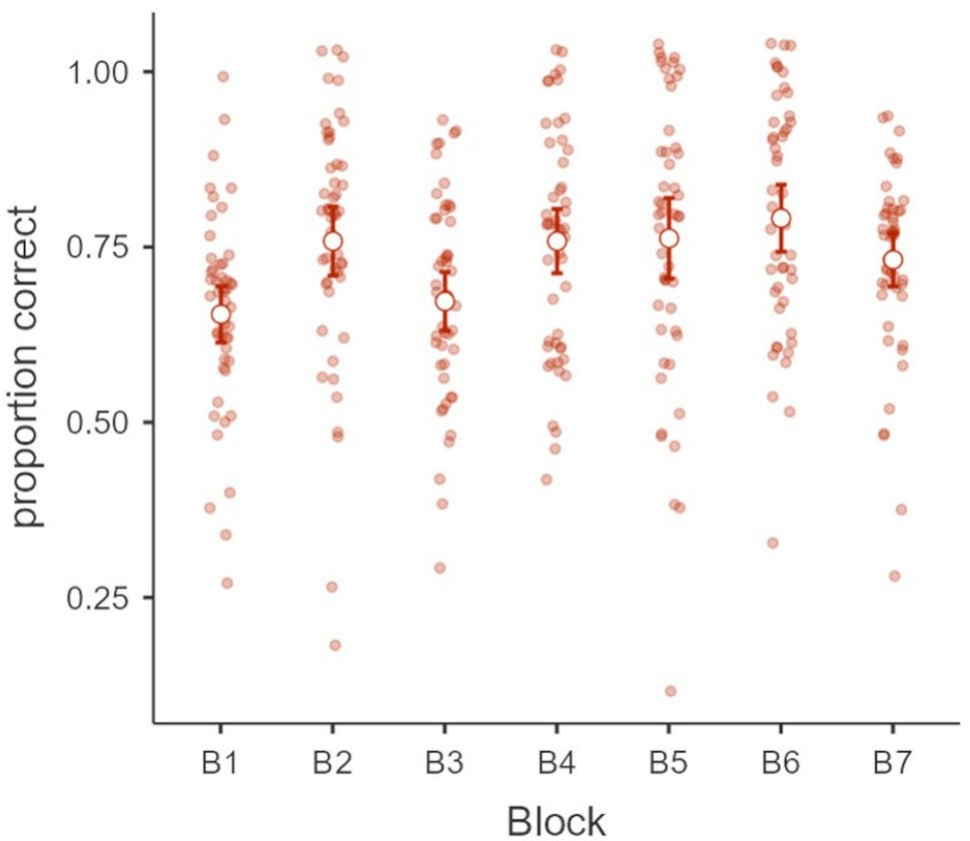
Mean proportion correct and 95% confidence intervals for the in the interim tests of retrieval practice

Mean performance in the cloze test used for retrieval practice increased across the learning blocks (cf. Fig. 2). A logarithmic function (proportion correct = 0.05 * ln(Learning Block) + 0.67) explained 44% of the variance in the data.

### Grammaticality judgment test

**Fig. 3:**
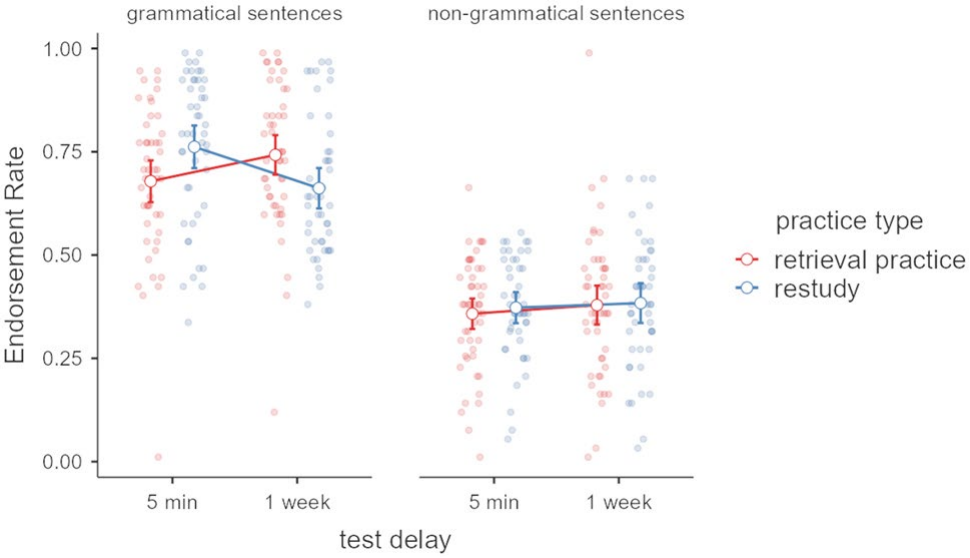
Mean endorsement rates and 95% confidence intervals for groups of practice type (retrieval practice vs. restudy) after 5 min and after 1 week. Lines illustrate that test delay is a repeated-measures variable. Dots represent individual data points

This test was provided after the 5-min and 1-week delay with new sets of sentences to purely assess transfer performance. Transfer performance (i.e., mean endorsement rate) was analyzed as a function of practice type and test delay, as can be seen in Fig. 3.

The results show that both groups of participants endorsed grammatical items more often as grammatical than non-grammatical items after both delays (see Fig. 3). This was confirmed by a significant main effect of sentence type, *F*_1,94_ = 245.68, *p* < 0.001, ${\upeta }_{\text{p}}^{2}$ = 0.72. In addition, we observed a significant two-way interaction between delay and practice type,* F*_1,94_ = 6.93, *p* = 0.010, ${\upeta }_{\text{p}}^{2}$ = 0.07, and a three-way interaction between delay, practice type, and sentence type, *F*_1,94_ = 23.26, *p* < 0.001, ${\upeta }_{\text{p}}^{2}$ = 0.20.

Follow-up, post-hoc tests conducted separately for grammatically correct and incorrect sentences revealed differential endorsement between the two groups for grammatical sentences (delay by practice-type interaction: *F*_1,94_ = 19.378, *p* < 0.001, ${\upeta }_{\text{p}}^{2}$ = 0.171), but not for non-grammatical sentences (delay by practice-type interaction: *F*_1,94_ < 1, *p* = 0.79). Subsequent analyses revealed that the restudy group endorsed significantly more grammatically correct sentences as grammatical than the retrieval-practice group after the short delay,* t*(94) = 2.29, *p* = 0.024, *d* = 0.47, but significantly fewer than the retrieval-practice group after a 1-week test delay,* t*(94) = − 2.34, *p* = 0.021, *d* = − 0.48.

**Fig. 4:**
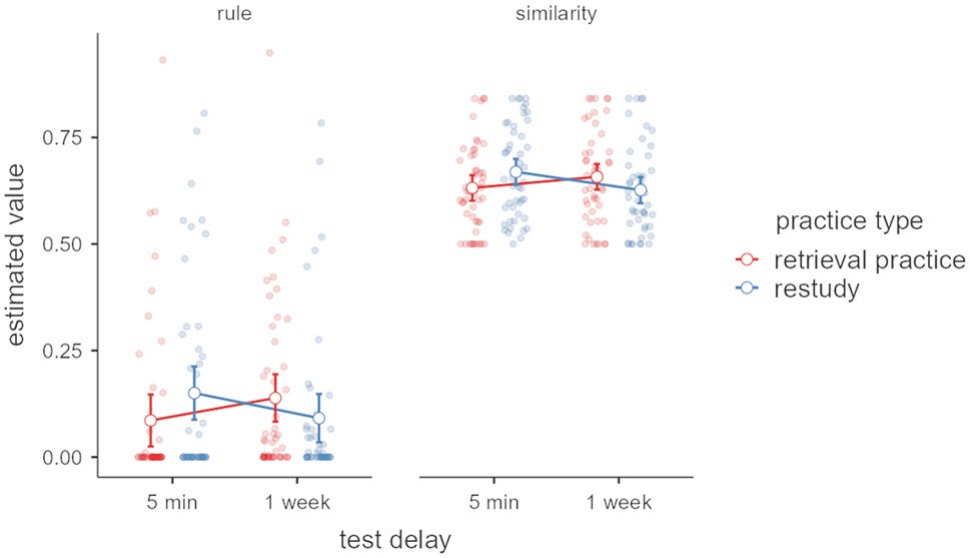
Parameter estimates and 95% confidence intervals derived from the formal hybrid model for individual ROCs for practice type (retrieval practice vs. restudy) after 5 min and 1 week. Lines illustrate that test delay is a repeated-measures variable. Dots represent individual data points

As previously suggested (Opitz & Hofmann, 2015), a hybrid model best accounts for empirical ROC data in the grammaticality judgment task. Model parameters reflecting rule-based and similarity-based learning were extracted from the best fitting model for each participant and subjected to a repeated-measures ANOVA with the within-subject factors parameter estimate (rule vs. similarity), delay (5 min vs. 1 week) and between-subject factor practice type (retrieval practice vs. restudy; see Fig. 4).

The results revealed a significant main effect of the factor parameter estimate, *F*_1,94_ = 784.06, *p* < 0.001, ${\upeta }_{\text{p}}^{2}$ = 0.89, indicating that participants relied more on similarity-based than on rule-based knowledge (Fig. 4). In addition, a significant two-way interaction between delay and practice type was observed, *F*_1,94_ = 22.34, *p* < 0.001, ${\upeta }_{\text{p}}^{2}$ = 0.19. To address the specific hypotheses, the same analyses were also conducted separately for both estimates. For the rule estimate, there was a significant interaction between delay and practice type, *F*_1,94_ = 8.05, *p* = 0.006, ${\upeta }_{\text{p}}^{2}$ = 0.08. Posthoc tests revealed that the restudy group relied less on rule-based knowledge after the long delay compared to the short delay, *t*(94) = -2.09, *p* = 0.039, *d* = -0.30, while the opposite pattern was marginally significant for the retrieval-practice group, *t*(94) = 1.98, *p* = 0.058, *d* = 0.28. A similar result was observed for similarity-based knowledge; again the restudy group relied less on similarity after a delay of 1 week compared to the short delay of 5 min, *t*(94) = -3.12, *p* = 0.002, while the retrieval-practice group seems to rely more on similarity, *t*(94) = 1.94, *p* = 0.055,* d* = 0.26.

### Cloze test

**Fig. 5:**
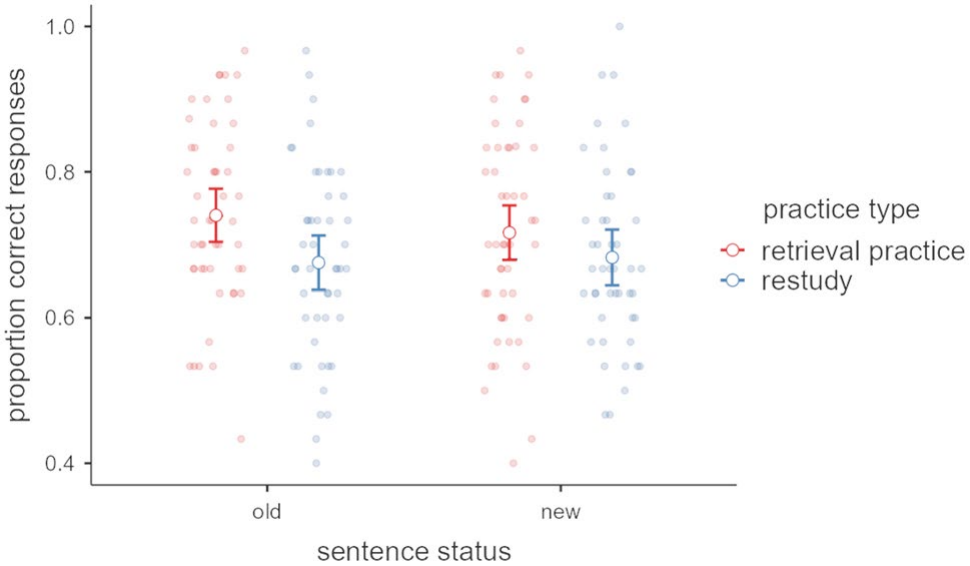
Proportion correct responses and 95% confidence intervals in the cloze test after 1 week for practice type (retrieval practice vs. restudy), separately for previously seen (old) versus not seen (new) sentences. Dots represent individual data points

This test assesses both memory performance in terms of the accuracy for previously seen (i.e., old) sentences and for new sentences as a function of practice type (see Fig. 5). The results revealed a main effect of practice type, *F*_1,94_ = 4.03, *p* = 0.048, ${\upeta }_{\text{p}}^{2}$ = 0.041, demonstrating that retrieval practice (mean proportion correct responses across old/new items = 0.728) outperformed restudy (mean proportion correct = 0.678). Crucially, neither the main effect of sentence status (old vs. new), *F*_1,94_ < 1, *p* = 0.404, ${\upeta }_{\text{p}}^{2}$ = 0.007, nor the interaction of both factors, *F*_1,94_ = 2.39, *p* = 0.125, ${\upeta }_{\text{p}}^{2}$ = 0.025, were significant, suggesting that both groups relied on rule knowledge rather than memory to complete the cloze test.

## Discussion

The aim of this study was to examine the effectiveness of retrieval practice during artificial language learning. Results revealed that restudy led participants to better discriminate grammatical from non-grammatical structures after a short delay compared to retrieval practice. Crucially, this restudy benefit on the immediate test was reversed into retrieval-practice benefit on the delayed transfer test after 1 week. This result pattern is in line with previous results demonstrating that participants who repeatedly studied a set of materials compared to those who took several tests tend to perform better in an immediate test but not in a delayed test (e.g., Roediger & Karpicke, 2006a, 2006b; Wheeler et al., 2003). It also demonstrates a substantial far-transfer effect of retrieval practice after 1 week. It is worth noting that test delay was manipulated as a within-subject variable in the present study. Note, however, that most other studies typically manipulate test delay as a between-subject manipulation, as any form of testing in the restudy group might enhance subsequent memory performance of the restudy in the final test (e.g., Nickl & Bäuml, 2023; Roediger & Karpicke, 2006a, 2006b). Despite this established effect of testing on memory performance, future studies should investigate whether the same applies to far-transfer effects as observed in the present study.

In line with the initial hypothesis, the ROC analysis revealed that retrieval practice facilitates rule-based learning. Compared to simply restudying the newly learned sentences, a cloze test to prompt retrieval practice might have encouraged participants to elaborate on the grammatical structure and consider which word class (e.g., a noun or a verb) would correctly complete the sentence fragment. This could have fostered the abstraction of grammatical rules and the generalization to new instances. Such rule knowledge is more efficient than rote memorization of learned sentences as a small set of rules is sufficient to describe the entire grammar of the artificial language but is much easier retained for a longer period than the huge number of potentially grammatically correct and incorrect sentences. The acquisition of the grammatical rules underlying the artificial language could also explain why participants in the retrieval-practice group correctly endorsed more grammatically correct sentences after a delay of 1 week compared to a delay of 5 minutes. It should be noted that compared to previous studies, the proportion of rule-based learning in the first session of the present study was relatively low (rule estimate = 0.12 across both groups vs. 0.41 in Opitz & Hofmann, 2015). It is conceivable that such a floor effect in the rule estimate could have blurred the transfer effects of retrieval practice. One likely explanation for this floor effect is related to differences in methodology to prior studies. In the study by Opitz and Hofmann (2015), feedback was provided during training, which led to more elaborate learning and promoted rule-based classifications during the final test. In contrast, in the present study, no performance feedback was provided at any stage of the study.

In addition, the view that rule-based learning underlies far transfer seems also to be supported by the results of the cloze test in the second session. This final memory test revealed similar performance for old (i.e., previously seen) and new sentences. This result pattern could be explained when performance is driven by rule-based rather than similarity-based learning. Although the finite number of sentences (70 sentences) learned in the present experiment could in principle be represented by mere memorization and, therefore, be accessible by similarity-based processes, this strategy does not allow the generalization to new and dissimilar instances from the nearly infinite body of potential grammatical sentences. Thus, it seems rather unlikely that similar performance for old and new sentences in the final cloze test is supported by similarity-based mechanisms. If this pattern of results were based on the memorization of learned instances (i.e., dominant similarity-based learning), better performance for old than new sentences would be expected. This is in apparent contrast to the present results.

Furthermore, the overall better performance of the retrieval-practice group compared to the restudy group in the cloze test also suggests that retrieval practice boosts rule-based rather than similarity-based learning. However, practice effects could not be ruled out because only the retrieval-practice group did the cloze test during the first session which might have contributed to the better performance due to transfer-appropriate processing (e.g., Morris et al., 1977).

Taken together, the present results support the notion of beneficial effects of retrieval practice also for transfer of learning. It seems plausible to assume that this retrieval-related benefit is driven by improved rule-based learning. Our interpretation that far transfer in the present study is promoted by rule-based learning of the grammar system is in agreement with the view that far transfer is supported by learning of underlying general principles (Pan & Pickard, 2018). It could be that retrieval practice forced learners to judge the role of particular words in a sentence and deduce their relationship at an abstract level. With repeated practice, the relationship between words could then be generalized beyond a specific word order and results in a set of rules describing the artificial grammar to a very high degree. These rules can be viewed as an organizational scaffold for the efficient judgment of new stimuli in the final test. That is, the incoming new stimulus rather than being compared with many exemplars stored in memory is now compared against a few grammatical rules. Furthermore, such a small set of rules is easier to retain over a long period in time—this could explain a similar performance of the retrieval-practice group after both delays, while performance of the restudy groups drops from the short to the long delay. This account of the present data is in line with abstractionist views supporting (far) transfer (see Barenberg et al., 2021; Barnett & Ceci, 2002; Pan & Pickard, 2018).

Alternatively, the powerful effect of retrieval practice in learning could be due to the introduction of desirable difficulties. Previous research suggests that learning methods and strategies that produce rapid learning and short-term benefits lead to poor long-term retention and in contrast, experiencing some degree of difficulty during learning can lead to higher levels of memory (Bjork & Bjork, 2020). The retrieval-effort hypothesis (see Pyc & Rawson, 2009) states that higher effort during successful retrieval leads to better retention of information over time. Therefore, the difference in grammatical judgment scores after 1 week favoring the retrieval-practice group could be due to the different levels of difficulty both groups experienced while learning, with a higher level of difficulty caused by the cloze test compared to restudying the same sentences. Although this memory account can explain differences between the retrieval practice and the restudy group in the grammaticality test, it could not readily explain the equal performance in the cloze test for old (i.e., previously seen) sentences and novel sentences for both groups.

It should be noted that there was a high attrition rate between both sessions of the study. 28 participants (about 19%) did not attempt the second session. However, the dropout rate was similar across both groups and all genders suggesting that data are missing at random. However, participants who dropped out showed a substantially lower endorsement rate for grammatical items in the first session compared to participants who completed both sessions (0.546 vs. 0.910). It is conceivable that participants who struggled completing the final test in the first session and had a feeling they had not done well (please note that no performance feedback was provided at any stage of the study) more often decided to drop out. This could have biased the results of the present study to limit the generalizability to participants with low learning performance (Bell et al., 2013).

## Conclusions

The current study replicated the finding that retrieval practice benefits long-term retention. Although there were no significant differences as function of practice type in the immediate test, the retrieval-practice group outperformed the restudy group after 1 week, which resulted largely from a performance drop in discrimination index scores for the restudy group. The results suggest that retrieval practice does not only benefit long-term retention, but could also foster rule learning, as opposed to memorization of the material. Thus, retrieval practice is also effective for learning complex materials and even more importantly, for transfer of learning crucial for modern educational practices (Richter et al., 2022). Given the far-ranging implications for the implementation of retrieval practice as a learning strategy in educational settings, further research is required exploring preconditions for the effectiveness of retrieval practice with complex materials like the role of initial comprehension (Rummer & Schweppe, 2022) or meaningful processing (Pan & Rickard, 2018) of the learning materials.

## Footnotes

1 Both terms “testing effect” and “retrieval practice effect” are synonymously used in the literature.
